# Impact of glucocorticoids on short-term and long-term outcomes in patients with relapsed/refractory multiple myeloma treated with CAR-T therapy

**DOI:** 10.3389/fimmu.2022.943004

**Published:** 2022-08-23

**Authors:** Xue Wang, Yuekun Qi, Hujun Li, Fengan Liu, Jiang Cao, Wei Chen, Ying Wang, Kunming Qi, Zhiling Yan, Feng Zhu, Zhenyu Li, Hai Cheng, Kailin Xu

**Affiliations:** ^1^ Department of Hematology, The Affiliated Hospital of Xuzhou Medical University, Xuzhou, China; ^2^ Blood Diseases Institute, Xuzhou Medical University, Xuzhou, China; ^3^ Jiangsu Key Laboratory of Bone Marrow Stem Cells, Xuzhou, China; ^4^ Xuzhou Medical University, Xuzhou, China

**Keywords:** chimeric antigen receptor T cell, glucocorticoids, relapsed/refractory, multiple myeloma, outcome

## Abstract

**Background:**

Glucocorticoids (GCs) are often used to treat cytokine release syndrome (CRS) and immune effector cell-associated neurotoxicity syndrome (ICANS). The effects of GCs on the efficacy of CAR-T cell treatment in relapsed/refractory multiple myeloma (RRMM) have not been fully established. We evaluated the impact of GCs on clinical outcomes of RRMM patients treated with CAR-T cells.

**Methods:**

This study involved RRMM patients treated with CAR-T cells at our center between June 2017 and December 2020. Patients were stratified into GC-used group (GC-group) and non-GC-used group (NGC-group). CRS or ICANS was graded on the basis of the American Society of Transplantation and Cellular Therapy consensus grading system. Response status was evaluated by the IMWG Uniform Response Criteria. The duration of response (DOR), progression-free survival (PFS), and overall survival (OS) were calculated.

**Result:**

A total of 71 patients were included in this study. In the NGC group (40 patients), 34 (85%) had responses to CAR-T cell therapy, including 16 (40%) stringent complete response (sCR), seven (17.5%) complete response (CR), five (12.5%) very good partial response (VGPR), and six (15%) partial response (PR). The overall response rate (ORR) and complete response rate (CRR) in the NGC group were 85% and 57.5%. In the GC group (31 patients), 29 (93.5%) had responses, including 11 (35.5%) sCR, nine (29%) CR, two (6.4%) VGPR, and seven (22.6%) PR. Differences in ORR and CRR between the two groups were insignificant. The dose, duration, and timing of GCs did not affect ORR and CRR. At a median follow-up of 28.2 months, the median PFS was 20.4 months (95% CI, 7.9 to 32.9) while the median OS was 36.6 months (95% CI, 25.9 to 47.2) for the GC group. The median PFS and OS for the NGC group were 13.7 months (95% CI, 8.8 to 18.6) and 27.5 months (95% CI, 14.1 to 41.0). There were no significant differences in either PFS or OS between the GC group and the NGC group. Differences in median DOR for the patients with CR or better in the GC group and NGC group were not significant (*p* = 0.17). Earlier, prolonged use and high dose of GCs were not associated with any effects on either PFS or OS. Additionally, GCs had no effects on CAR-T cell proliferation.

**Conclusion:**

Administration of GCs, dose, timing, and duration does not influence the clinical efficacy of CAR-T cells in RRMM in this study.

## Introduction

Anti–B cell maturation antigen (BCMA) CAR-T cell therapy has attained encouraging efficacy in treating relapsed/refractory multiple myeloma (RRMM) (1–6). However, CAR-T cell therapy-associated immunological toxicity remains a major clinical challenge (7). Although glucocorticoids (GCs) can mitigate severe adverse effects, the expansion and persistence of CAR-T cells may also be sacrificed, which adversely affect clinical outcomes (8–10). Clinical trial data reported are very confusing about the role of GCs for the management of CRS and/or immune effector cell-associated neurotoxicity syndrome (ICANS). Early studies showed that GCs might not impact the efficacy of CAR-T cells for B-cell acute lymphoblastic leukemia (B-ALL) (11). Recent studies reported that high cumulative dose and prolonged application of GCs negatively affected overall survival (OS) in large B-cell lymphoma (LBCL) (12). However, the effect of GCs on CAR-T cell efficacy in RRMM has not been established.

It is necessary to clarify whether the use of GCs and their dose, timing, and duration could impact clinical outcomes in clinical trials for RRMM. Herein, we carried out a retrospective analysis to investigate the impacts of GCs on outcomes of CAR-T cell therapy in RRMM.

## Methods

### Patient selection

This retrospective investigation was approved by the ethics committee of the Affiliated Hospital of Xuzhou Medical University. RRMM patients treated with CAR-T cells at our center between June 2017 and December 2020 were included in this study (ChiCTR1900026219, ChiCTR-OIC-17011272). All patients were infused with anti-BCMA CAR-T cells alone or combined with anti-CD19 CAR-T cells. CAR structures targeting CD19 and BCMA were as described previously (13, 14). The data cutoff for follow-up was 30 November 2021.

### CRS and ICANS management

CRS or ICANS was graded according to the ASTCT consensus grading system. Fever is defined as a temperature ≥38°C after CAR-T cell infusion according to ASTCT consensus (15). Dexamethasone or methylprednisolone was administrated when antipyretics (aspirin-DI-lysine, 900 mg, each time, up to 3,600 mg per day) failed to resolve continuing fever (≥38°C) and ICANS.

### Clinical outcome and follow-up

The IMWG Uniform Response Criteria for Multiple Myeloma were used to evaluate clinical response (16). Patients were monitored at 2 weeks, 1 month, 2 months, 3 months, and 6 months after CAR-T cell infusion. After 1 year, regular follow-up was conducted every 6 months. Further assessments were as previously reported (14).

### Evaluation of laboratory parameters

Complete blood counts (CBC) and ferritin, IL-6, and CRP levels were assessed before lymphodepletion, on the day of CAR T-cell infusion, and at intervals following CAR-T cell infusion. CAR-T cell counts in peripheral blood were assessed by flow cytometry (FCM) (17, 18).

### Statistical methods

Absolute number and percentage were used to describe categorical variables. Mann–Whitney U-test was used in the case of non-parametric data. Kaplan–Meier analysis was used to estimate the duration of response (DOR), progression-free survival (PFS), and overall survival (OS). A comparison of overall response rate (ORR) and complete response rate (CRR) between the GC-used group (GC-group) and non-GC-used group (NGC-group) was analyzed using Fisher’s exact test. All statistical analyses were performed using the IBM SPSS 25.0 software, and two-sided *P* values less than 0.05 were considered to be statistically significant.

## Results

### Patient characteristics

A total of 71 patients were stratified into GC group and NGC group. The median age was 57 years (range, 29 to 70), and 12.7% of patients were older than 65 years. Fourteen patients in the NGC group received auto-hematopoietic stem cell transplantation (auto-HSCT) while four patients in the GC group received auto-HSCT (*p* = 0.03). Other baseline characteristics were not remarkably different between the two groups (**Table 1**).

**Table 1:** Baseline characteristics and association with GC use.

	Total Median	GC-group	NGC-group	*P*
**No. (%)**	71	31 (43.7)	40 (56.3)	
**Male, no. (%)**	46 (64.8)	18 (58.1)	28 (70.0)	0.30
**Age, median (range)**	57 (29-70)	57 (29-70)	57 (40-70)	0.34
**Types, no. (%)**				
**IgG**	31 (43.7)	12 (38.7)	19 (47.5)	0.46
**IgA**	15 (21.2)	6 (19.4)	9 (22.5)	0.75
**IgD**	8 (11.3)	4 (12.9)	4 (10.0)	0.72
**Light chain**	13 (18.3)	8 (25.8)	5 (12.5)	0.15
**Non-secretory**	4 (5.6)	1 (3.2)	3 (7.5)	0.63
**Time from diagnosis to enrolment, months**	30 (3-170)	26 (5-165)	31 (3-170)	0.19
*ISS, stage III, no. (%)	37 (52.1)	15 (48.4)	22 (55.0)	0.58
**†High tumor burden, no. (%)**	15 (21.1)	8 (25.8)	7 (17.5)	0.40
**‡High-risk cytogenetics, no. (%)**	18 (25.4)	10 (32.3)	8 (20.0)	0.24
**Previous auto-HSCT, no. (%)**	18 (25.3)	4 (12.9)	14 (35.0)	0.03
**Previous therapy lines**	4 (2-17)	3 (2-11)	4 (2-17)	0.07
**Complete blood count**				
**Platelet count (×10^9^/L)**	120 (17-329)	140 (17-329)	119 (23-259)	0.59
**White blood cell count (×10^9^/L)**	3.8 (1.1-9.9)	4.1 (1.1-9.9)	3.7 (1.6-6.2)	0.20
**Hemoglobin (g/L)**	96 (47-158)	105 (47-149)	93 (56-158)	0.37
**Absolute lymphocyte count (×10^9^/L)**	1.1 (0.4-4.7)	1.2 (0.1-4.7)	1.0 (0.4-3.1)	0.25
**Absolute neutrophil count (×10^9^/L)**	2.1 (0.4-6.7)	2.3 (0.5-6.7)	1.9 (0.4-3.7)	0.43
**Acute-phase proteins**				
**CRP (mg/L)**	4.5 (0-99.0)	4.2 (0-99.0)	4.5 (1.0-83.0)	0.67
**IL-6 (pg/mL)**	5.9 (1.5-50.8)	7.7 (1.5-44.4)	5.7 (1.5-50.8)	0.46
**Ferritin (ng/mL)**	483.5 (98.4-4489.0)	450.4 (107.7-4489.0)	485.0 (98.4-2000.0)	0.69

*ISS, International Staging System.

†High tumor burden was defined as at least 50% plasma cells in bone marrow.

**‡**High-risk cytogenetics: presence of del(17p) and/or translocation t (4, 14) and/or translocation t (14, 16).

Auto-HSCT, auto-hematopoietic stem cell transplantation.

Two-sided P values were calculated using the Mann–Whitney U test for continuous variables and Pearson chi-square or Fisher’s exact test for categorical variables.

### Management of CRS and ICANS

**Figure 1:**
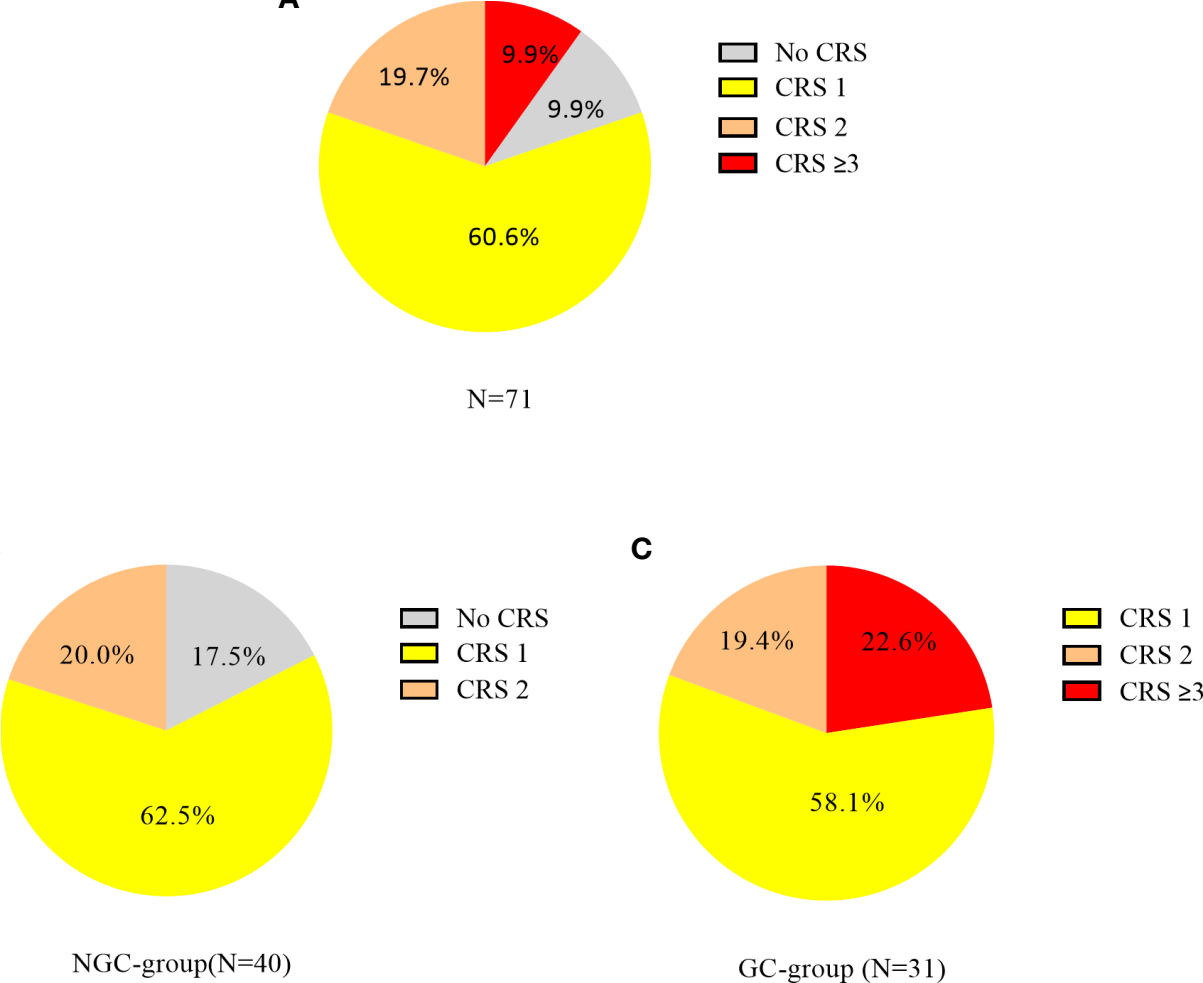
Cytokine release syndrome (CRS) incidence and severity in patients with CAR-T cell therapy. CRS was graded according to the ASTCT consensus grading system. **(A)** CRS incidence in all patients; **(B)** CRS incidence in the NGC group. **(C)** CRS incidence in the GC group.

CRS occurred in 64 (90.1%) of 71 patients, 43 (60.6%) had grade 1, 14 (19.7%) had grade 2, and 7 (9.9%) had grade ≥3 CRS. In the NGC group (n = 40), 25 patients had grade 1, 8 had grade 2, and 7 had no CRS. In the GC group (n = 31), 18 patients had grade 1, 6 had grade 2, and 7 had grade ≥ 3 CRS (**Figure 1**). ICANS occurred in 5 (7.0%) of 71 patients, and 2 (2.8%) patients had grade ≥3 ICANS. In the NGC group, 8/40 patients with grade 2 CRS required the use of oxygen by low-flow nasal cannula. In the GC group, 24/31 patients received dexamethasone (5–10 mg, q6h) and 7 received methylprednisolone (1–2 mg/kg/day). The median cumulative GC dose was equivalent to 35 mg of dexamethasone (range, 5 to 450) and the median duration of GCs was 3 days (range, 1 to 34). The reason for the prolonged use of GCs was persistent symptom of CRS or ICANS. Eleven (35.5%) of 31 patients received GCs within the first 7 days post CAR-T cell infusion and 20 (64.5%) beyond day 7.

### GCs and ORR or CRR

Patients achieved the best response within a median time of 70 days (range 14–207 days). In the NGC group, responses were observed in 34 patients, and ORR was 85%, including 16 (40%) stringent complete response (sCR), seven (17.5%) complete response (CR), five (12.5%) very good partial response (VGPR), and six (15%) partial response (PR). In the GC group, responses were observed in 29 patients, and ORR was 93.5%, including 11 (35.5%) sCR, nine (29%) CR, two (6.4%) VGPR, and seven (22.6%) PR. Differences in ORR and CRR between the NGC group and GC group were insignificant (**Table 2**). Considering the difference in the proportion of patients who received auto-HSCT between the two groups, Cochran’s and Mantel–Haenszel statistics was performed. There was no difference in ORR and CRR between the two groups. We also investigated whether GC doses were associated with ORR and CRR. According to cumulative dose, 31 patients in the GC group were divided into two subgroups: high-dose GC group (dexamethasone-equivalent dose >35 mg, HGC-group) and low-dose GC group (dexamethasone-equivalent ≤35 mg, LGC group). No significant difference in ORR and CRR was observed between the high-dose group and low-dose group (**Table 2**). To clarify whether the timing of GCs could affect the response, patients in the GC group were divided into two groups, 11 of 31 patients received GCs within 7 days (≤7-day group) and 20 beyond 7 days (>7-day group). There was no correlation between timing of GCs and responses (**Table 3**).

**Table 2:** Association between GCs and responses.

Response category	GC use		*P*	GC dose		*P*
	GC group (n=31)	NGC group (n=40)		HGC group (n=15)	LGC group (n=16)	
**Overall response**						
No. with response	29	34		14	15	
Rate—% (95% CI)	93.5 (78.6-99.2)	85.0 (70.2-94.3)	0.45	93.3 (68.1-99.8)	93.8 (69.8-99.8)	1.00
**Best overall response, no. (%)**						
Complete response or better	20 (64.5)	23 (57.5)	0.63	10 (66.7)	10 (62.5)	1.00
Complete response	9 (29.0)	7 (17.5)		6 (40.0)	3 (18.8)	
Stringent complete response	11 (35.5)	16 (40.0)		4 (26.7)	7 (43.8)	
Very good partial response or better	22 (71.0)	28 (70.0)	0.93	11 (73.3)	11 (68.9)	1.00
Very good partial response	2 (6.5)	5 (12.5)		1 (6.7)	1 (6.3)	
Partial response	7 (22.6)	6 (15.0)		3 (20.0)	4 (25.0)	

Response status was determined by IMWG Uniform Response Criteria for Multiple Myeloma. P values were calculated with the chi-square test.

HGC-group, cumulative dexamethasone-equivalent dose >35 mg; LGC-group, cumulative dexamethasone-equivalent dose ≤35 mg.

**Table 3:** Association between duration, the timing of GCs, and responses.

Response category	Timing of GC use		*P*	Duration of GC use		*P*
	≤7-day (n=11)	>7-day (n=20)		≤3-day (n=16)	>3-day (n=15)	
**Overall response**						
No. with response	11	18		15	14	
Rate—% (95% CI)	100 (71.5-100.0)	90.0 (68.3-98.8)	0.53	93.8 (69.8-99.8)	93.3 (68.1-99.8)	1.00
**Best overall response, no. (%)**						
Complete response or better	8 (72.7)	12 (60.0)	0.70	10 (62.5)	10 (66.7)	1.00
Complete response	3 (27.2)	6 (30.0)		4 (25.0)	5 (33.3)	
Stringent complete response	5 (45.5)	6 (30.0)		6 (37.5)	5 (33.3)	
Very good partial response or better	10 (90.9)	12 (60.0)	0.11	11 (68.9)	11 (73.3)	1.00
Very good partial response	2 (18.2)	0		1 (6.3)	1 (6.7)	
Partial response	1 (9.1)	6 (30.0)		4 (25.0)	3 (20.0)	

Response status was determined by IMWG Uniform Response Criteria for Multiple Myeloma.

≤7-day group, received GCs within 7 days post CAR-T cell infusion; >7-day group, received GCs beyond 7 days; ≤3-day group, duration of GCs uses 3 days or less than 3 days; >3-day group, duration of GC uses more than 3 days.

### GCs and DOR

**Figure 2:**
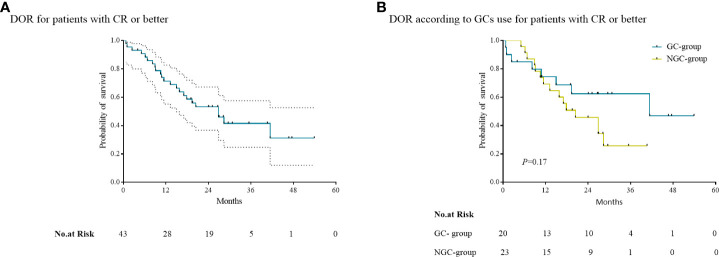
Kaplan–Meier curves of the duration of response (DOR). **(A)** The median DOR for patients with CR or better. **(B)** The median DOR for patients according to the use of GCs in patients with CR or better.

The median DOR for patients with CR or better was 26.8 months (95% CI, 16.7 to 36.9) (**Figure 2A**) . In the GC group, the median DOR for 20 patients having CR or better was not reached. In the NGC group, the median DOR for 23 patients having CR or better was 20.5 months (95% CI, 10.7 to 30.2) (**Figure 2B**). Differences in median DOR for the patients with CR or better in the GC group and NGC group were not significant (*p* = 0.17).

### GCs and OS or PFS

**Figure 3:**
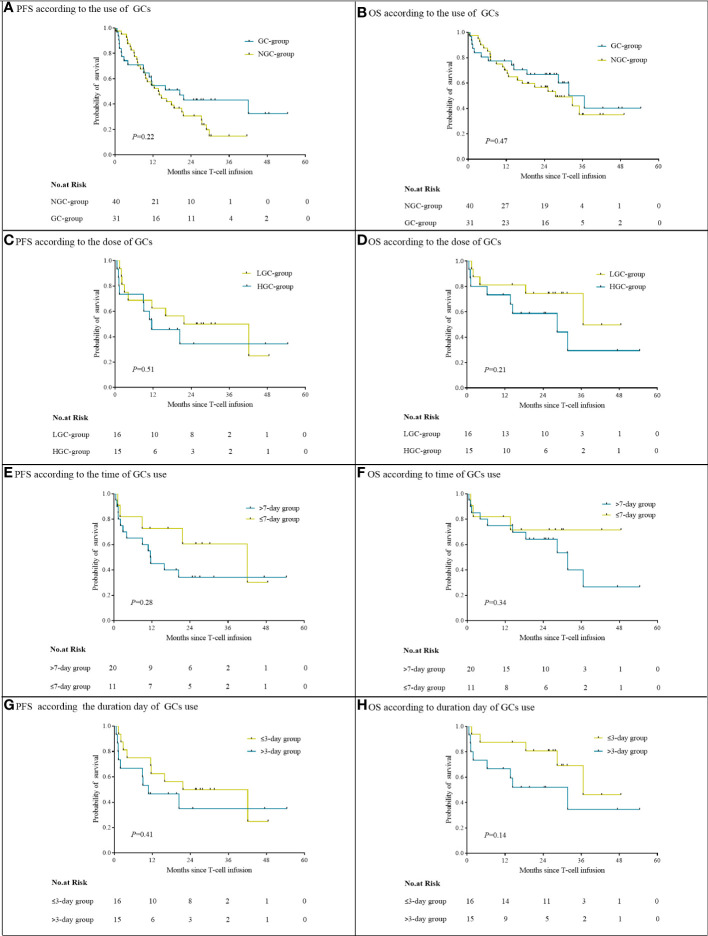
Progression-free survival and overall survival, according to use of GCs **(A, B)**, cumulative GC dose **(C, D)**, timing **(E, F)**, and duration **(G, H)**.

At a median follow-up of 28.2 months, the median PFS in the GC group was 20.4 months (95% CI, 7.9 to 32.9) and the median OS was 36.6 months [95% CI, 25.9 to 47.2]. The median PFS and OS in the NGC group were 13.7 months (95% CI, 8.8 to 18.6) and 27.5 months (95% CI, 14.1 to 41.0). There were no significant differences in either PFS or OS between the NGC group and GC group (**Figures 3A, B**). The median PFS and OS in the HGC group were 11.5 months (95% CI, 0.1 to 22.9) and 28.4 months (95% CI, 0.2 to 58.4). The median PFS in the LGC group was 21.7 months (95% CI, 1.2 to 42.2), and the median OS was not reached. No significant difference was observed in PFS and OS between the HGC group and LGC group (**Figures 3C, D**). We did not observe a remarkable association between the time or duration of GC use and survival (**Figures 3E–H**).

### GCs and the proliferation of CAR-T cell

**Figure 4:**
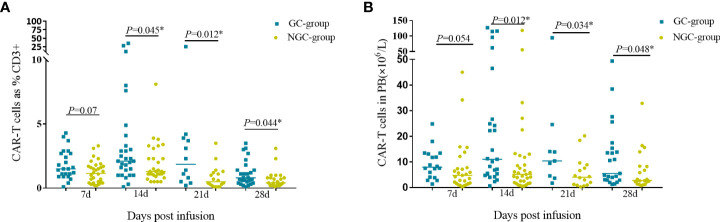
CAR-T cell expansion and persistence in peripheral blood. Proliferation of CAR-T cells in peripheral blood was assessed by flow cytometry on days 7, 14, 21, and 28. **(A)**. The percentage of CAR-T cells in the peripheral blood. **(B)** Counts of CAR-T cells in the peripheral blood. *Represents for statistically different (P < 0.05).

CAR-T cells started to proliferate within 7 days in both GC group and NGC group. The percentage and counts of CAR-T cells reached a peak at day 14 then decreased gradually. The median percentage and CAR-T cell counts in the GC group were higher than those in the NGC group at indicated time points (days 7, 14, 21, and 28 post CAR-T infusion) (**Figure 4**). There was a remarkable difference in median percentage and counts of CAR-T cells between the GC group and NGC group (**Figure 4**).

## Discussion

GCs are effective in reducing CAR-T-related adverse events. There are theoretical concerns about whether the use of GCs will affect the expansion of CAR T cells as well as impair their efficiency in killing tumor cells. Limited studies exploring the impact of GCs on clinical outcomes of CAR-T cell therapy are mainly from clinical trials for B-ALL and LBCL (19–21). To the best of our knowledge, this is the first retrospective study to investigate the effects of cumulative dose, timing, and duration of GCs on the efficacy of CAR-T cells in RRMM.

We found no relation between GCs and ORR or CRR, contrary to earlier reports. High dosages of GCs are effective for the treatment of CRS, generating concerns about the effectiveness of CAR-T. Our research exhibited that the use of GCs had no effects on the efficacies of CAR-T therapy, including ORR and CRR, irrespective of cumulative dosage, timing, or duration of GC use. Liu et al. (20) recently discovered that low-dose GCs for a short period of time (average 4 days) had no effect on outcomes of CAR-T cell therapy. Our findings were also consistent with other clinical trials. There are two largest investigations conducted by Strati et al. (12) and Neelapu et al. (19), respectively, none of which showed an association between GCs and ORR or CRR.

The association between GCs and long-term survival was also evaluated. We established that the dose, duration, and timing of GCs use did not impact PFS or OS. In another study, high dose, timing, and prolonged use of GCs were linked to shorter PFS and OS. We compared the differences between these two studies and found that the study by Strati et al. reported negative results from the tendency to use high doses of GCs for a long period (median cumulative dexamethasone-equivalent dose and duration were 186 mg and 9 days, respectively). However, in our study, the median cumulative GC dose was equivalent to 35 mg of dexamethasone (range, 5 to 450) while the median duration of GCs was 3 days (range, 1 to 34). Consistent with our findings, Sesques et al. and Nastoupil et al. did not find any correlation between GC use and PFS (22, 23). Additionally, Holtzman et al. (24) reported that a much longer and higher cumulative dose of GC treatment did not impact PFS or OS in patients with ICANS. In this study, patients with ICANS required GC treatment, with a median total GC dose equal to 221 mg of dexamethasone given for a median of 12.5 days. In contrast to earlier studies, neither the dosage nor the duration of GCs was correlated with a shorter PFS or OS.

Previous clinical studies have shown that for different primary diseases, the application of GCs has different effects on the clinical outcomes (12, 22). Early studies showed that GCs might impact the efficacy of CAR-T cells for B-ALL (21). Two recent investigations exhibited that early treatment with GCs for CRS or ICANS had no influence on the antitumor potency of CD19 CAR-T cells and the potential mechanisms have been postulated (25–27). Our findings illustrated that GCs, even high-dose GCs, had no effect on CAR-T cell growth. It is possible that our results differ from prior findings because we utilized high-dose GCs for a short period of time, while they were used for much longer. Limitations of our study include its retrospective nature and the small sample size, which prevented use of a propensity score matching analysis. Early GC use in CAR-T toxicity control is being explored (28), and although more prospective trials are required, our results may relieve anxiety regarding GC use in CRS treatment.

## Conclusions

In summary, although limited by small sample size and likely patient selection bias, GC use was not associated with lower CRR or ORR and shorter PFS, or OS in RRMM patients treated with CAR-T cells. Moreover, GCs had no detectable influence on patient treatment outcomes, regardless of duration or cumulative dosage in RRMM patients.

## Data availability statement

The original contributions presented in the study are included in the article/supplementary material. Further inquiries can be directed to the corresponding authors.

## Ethics statement

The studies involving human participants were reviewed and approved by Ethics committee of the Affiliated Hospital of Xuzhou Medical University. The patients/participants provided their written informed consent to participate in this study.

## Author contributions

HC and KX designed the research study. All investigators and their respective research teams recruited and followed up the patient. XW, YQ, and HL analyzed the data and wrote the first draft of the paper. All authors reviewed and critically edited the manuscript. All authors contributed to the article and approved the submitted version.

## Funding

This work is supported by the National Natural Science Foundation of China (81930005, 82070127, 81871263).

## Conflict of interest

The authors declare that the research was conducted in the absence of any commercial or financial relationships that could be construed as a potential conflict of interest.

## Publisher’s note

All claims expressed in this article are solely those of the authors and do not necessarily represent those of their affiliated organizations, or those of the publisher, the editors and the reviewers. Any product that may be evaluated in this article, or claim that may be made by its manufacturer, is not guaranteed or endorsed by the publisher.
